# Transmission of Equine Influenza Virus during an Outbreak Is Characterized by Frequent Mixed Infections and Loose Transmission Bottlenecks

**DOI:** 10.1371/journal.ppat.1003081

**Published:** 2012-12-20

**Authors:** Joseph Hughes, Richard C. Allen, Marc Baguelin, Katie Hampson, Gregory J. Baillie, Debra Elton, J. Richard Newton, Paul Kellam, James L. N. Wood, Edward C. Holmes, Pablo R. Murcia

**Affiliations:** 1 Medical Research Council-University of Glasgow Centre for Virus Research, Institute of Infection, Inflammation and Immunity, College of Medical, Veterinary and Life Sciences, University of Glasgow, Glasgow, United Kingdom; 2 Disease Dynamics Unit, Department of Veterinary Medicine, University of Cambridge, Cambridge, United Kingdom; 3 Immunisation, Hepatitis and Blood Safety Department, Health Protection Agency, London, United Kingdom; 4 Centre for Mathematical Modelling of Infectious Diseases, London School of Hygiene and Tropical Medicine, London, United Kingdom; 5 Boyd Orr Centre for Population and Ecosystem Health, Institute for Biodiversity, Animal Health and Comparative Medicine, University of Glasgow, Glasgow, United Kingdom; 6 Wellcome Trust Sanger Institute, Wellcome Trust Genome Campus, Hinxton, Cambridge, United Kingdom; 7 Animal Health Trust, Centre for Preventive Medicine, Lanwades Park, Newmarket, United Kingdom; 8 Center for Infectious Disease Dynamics, Department of Biology, The Pennsylvania State University, University Park, Pennsylvania, United States of America; 9 Fogarty International Center, National Institute of Health, Bethesda, Maryland, United States of America; Pasteur Institute, France

## Abstract

The ability of influenza A viruses (IAVs) to cross species barriers and evade host immunity is a major public health concern. Studies on the phylodynamics of IAVs across different scales – from the individual to the population – are essential for devising effective measures to predict, prevent or contain influenza emergence. Understanding how IAVs spread and evolve during outbreaks is critical for the management of epidemics. Reconstructing the transmission network during a single outbreak by sampling viral genetic data in time and space can generate insights about these processes. Here, we obtained intra-host viral sequence data from horses infected with equine influenza virus (EIV) to reconstruct the spread of EIV during a large outbreak. To this end, we analyzed within-host viral populations from sequences covering 90% of the infected yards. By combining gene sequence analyses with epidemiological data, we inferred a plausible transmission network, in turn enabling the comparison of transmission patterns during the course of the outbreak and revealing important epidemiological features that were not apparent using either approach alone. The EIV populations displayed high levels of genetic diversity, and in many cases we observed distinct viral populations containing a dominant variant and a number of related minor variants that were transmitted between infectious horses. In addition, we found evidence of frequent mixed infections and loose transmission bottlenecks in these naturally occurring populations. These frequent mixed infections likely influence the size of epidemics.

## Author Summary

Influenza A viruses (IAVs) are major pathogens of humans and animals. Understanding how IAVs spread and evolve at different scales (individual, regional, global) in natural conditions is critical for preventing or managing influenza epidemics. A vast body of knowledge has been generated on the evolution of IAVs at the global scale. Additionally, recent experimental transmission studies have examined the diversity and transmission of influenza viruses within and between hosts. However, most studies on the spread of IAVs during epidemics have been based on consensus viral sequences, an approach that does not have enough discriminatory power to reveal exact transmission pathways. Here, we analyzed multiple within-host viral populations from different horses infected with equine influenza virus (EIV) during the course of an outbreak in a population within a confined area. This provided an opportunity to examine the genetic diversity of the viruses within single animals, the transmission of the viruses between each closely confined population within a yard, and the transmission between horses in different yards. We show that individual horses can be infected by viruses from more than one other horse, which has important implications for facilitating segment reassortment and the evolution of EIV. Additionally, by combining viral sequencing data and epidemiological data we show that the high levels of mixed infections can reveal the underlying epidemiological dynamics of the outbreak, and that epidemic size could be underestimated if only epidemiological data is considered. As sequencing technologies become cheaper and faster, these analyses could be undertaken almost in real-time and help control future outbreaks.

## Introduction

Studying the evolution of influenza A viruses (IAVs) across different scales – from the individual to the global population – is critical for understanding the risk of cross-species transmissions and the potential for emergence of novel pandemic viruses. Time-informed phylogenetic approaches have been instrumental in understanding the evolutionary origin of recent pandemic strains [1], and experimental studies of naturally transmitted IAVs have revealed the patterns of genetic variation at the level of single hosts as well as the inter-host transmission of viral variants [2], [3]. To date, however, few studies have achieved sufficiently dense sampling during a naturally occurring outbreak to integrate epidemiological processes with evolution at the scale of individual hosts [4].

Equine influenza virus (EIV) is the aetiological agent of equine influenza (EI), an important disease of the horse. Two EIV subtypes have been detected: H7N7, now believed to be extinct [5], [6], and the currently circulating H3N8 subtype, which is distributed across most of the world as a result of the international movement of horses [7]–[10]. Currently, two clades of EIV circulate worldwide, and are denoted Florida Clades 1 and 2 [11], [12]. In the early 2000's, EIV jumped the species barrier and emerged as a novel respiratory virus in dogs, canine influenza virus (CIV) [13].

Newmarket, United Kingdom, is a town with a high density of thoroughbred horses. These animals are kept in individual stables, in yards that hold between ∼20 and 250 horses each. Yards are geographically very close to each other (Figure S1). In some cases, horses are in modern barn systems with a shared airspace for <∼30 animals, while in others they are in more traditional single stable system with no shared airspace. Each horse goes out to train once daily for approximately one hour (usually not on Sundays) in ‘strings’ that just consist of animals from their yard. Hence, other than passing on the paths or roads to and from the training areas, there is little mixing of horses among yards (see Fig. 2 in [14] for more detail). Despite high vaccination coverage (vaccination of racehorses is mandatory in the UK) Newmarket has periodically experienced EI outbreaks. In the spring of 2003 a large EI outbreak affected recently vaccinated horses in yards throughout the town. This outbreak was part of a larger epidemic that affected horses elsewhere in the UK and also in Europe [14]–[16]. Unlike previous localized EI outbreaks, the 2003 outbreak saw infections in a high number of yards in Newmarket.

Like other RNA viruses, intra-host populations of EIV are typically large and experience high rates of mutation [12], [17]. Transmission studies have shown that intra-host IAV populations display relatively high levels of genetic variation and relatively loose transmission bottlenecks, such that multiple viral variants are passed between animals at transmission [2], [3], [18]. Because multiple variants are transmitted between hosts, these minor variants could be potentially used to reconstruct the network of inter-host transmission during an outbreak. In turn, such a network could provide important insights into the mechanisms that shape viral diversity during localized epidemics and their impact at the global scale. Accurate identification of inter-horse transmission pathways will depend on the extent and structure of intra-yard and intra-host viral genetic variation, as well as the size of the transmission bottlenecks [19]. Although phylogenetic methods have been very informative for revealing key aspects of the evolution and spread of viruses [20], [21], they have limitations for inferring transmission trees from densely sampled outbreaks where population consensus sequences exhibit extreme similarity and where samples include both ancestral and descendent isolates. Indeed, recently developed graph approaches provide an alternative method to reconstruct transmission trees from molecular data and accompanying sampling dates and locations [22].

Here, we examined intra-host EIV genetic variation from horses affected during the 2003 EI outbreak that took place in Newmarket. We used both phylogenetic and graph approaches to infer the pathways of virus transmission. We also determined the transmission potential of EIV during the course of the outbreak by combining gene sequence and epidemiological data.

## Results

### Spatio-temporal patterns of influenza infection during the outbreak

**Figure 1:**
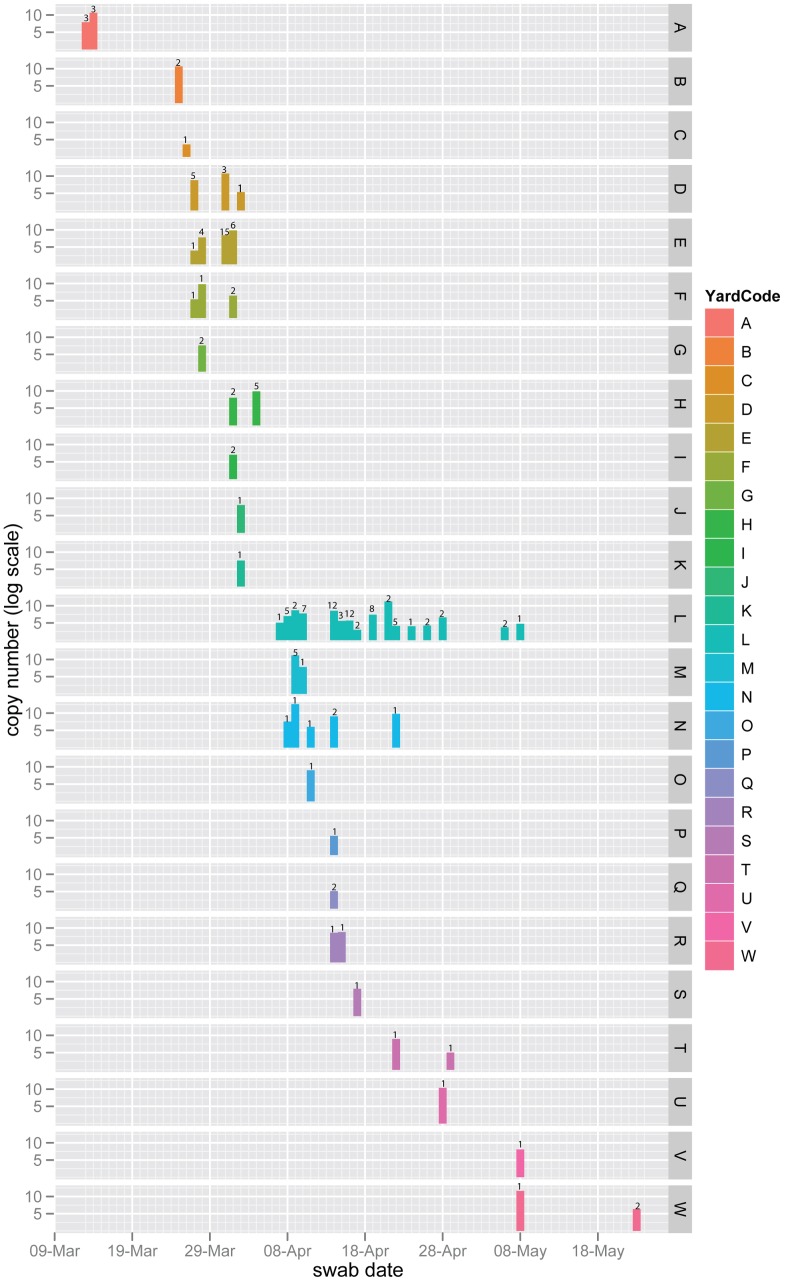
Daily cumulative viral shedding load per yard. Vertical bars represent the sum of viral copy numbers estimated by real-time PCR from all horses sampled on the same day on a natural log scale. The numbers above the bars represent the number of horses. These data were derived from nasal swabs obtained from 120 different horses for which yard location and sampling date were known (n = 154). Yards (A to W) are color-coded and the date in which the sample was taken is shown on the x-axis.

Nasal swabs from horses were obtained from 19 of the 21 (90%) yards in Newmarket infected during the outbreak (March 13^th^ to May 8^th^ 2003) as well as three yards located outside of Newmarket (yards O, T and W). Details about the number of horses in training per yard, as well as the yard location and date of first diagnosis can be found in [16]. We estimated the size of within-host viral populations by qPCR. Virus circulation was particularly high in Newmarket between the 7^th^ and the 29^th^ of April when 11 yards were concurrently infected (Figure 1). We combined these data with previously available data from a rapid diagnostic ELISA-based test [14] to determine the order in which the yards became infected based on the recorded date of each sample. Our results show that the order of infection was A>B>C>D+E+F+G>H+I>J+K followed by all the remaining yards (Figure 1 and Figure S1).

Horses were sampled, as described in [16], typically when affected with clinical respiratory disease during the outbreak. We obtained within-host viral sequences from samples that exhibited viral populations of sufficient size (measured by qPCR). In total, we sequenced viral populations from 50 horses, representing yards A to W. This subset of samples spanned the outbreak peak as they were obtained between March 13^th^ and May 8^th^. Viral populations from three horses (L25, L27, L42) were sampled at two separate time points. We geo-located 48 horses with viral sequences to their training yards (45 in Newmarket) (Figure S1).

### Naturally infected horses exhibit high levels of within-host viral diversity

We sequenced 2361 clones of the hemagglutinin 1 gene (HA1) derived from intra-host samples (a total of 2,131,983 nucleotides, GenBank accession numbers HE967958 to HE970318, Dataset S1) that spanned the first 903 nucleotides (nt) of HA1. This region of HA1 includes all the putative antigenic sites and the receptor-binding domain. To define mutations in our data set we used as a reference a consensus sequence from an isolate obtained during the outbreak (A/equine/Newmarket/5/2003, GenBank accession number FJ375213.1). We detected 493 different mutations, of which 321 were found in individual horses and not shared with others. The estimated mutation frequency ranged from 2.3E^−4^ to 7.5E^−4^ mutations per nucleotide site (when only unique mutations were considered or all mutations were assumed to have originated *de novo*, respectively). For these data, the mean pairwise distance per sample ranged from 0.0002 to 0.0025 and the mean d_N_/d_S_ per sample varied between 0.14 and 19.89. The estimated d_N_/d_S_ for the data set as a whole was 0.89 (95% CI = 0.80, 0.97), indicative of weak purifying selection over the eight-week period, and no sites were documented to be under significant positive selection.

Overall, we observed mutations at 455 nucleotide sites in HA1, of which 161 were synonymous and 332 non-synonymous. Interestingly, 659 sequences carried a specific non-synonymous mutation at site 230, and 31 sequences also carried the synonymous mutations C69T and A384T. Site 230 is polymorphic at the epidemiological-scale (91% G, 9% A), as is site 69 (97.9% C, 1.4% T, 0.7% A), while all other epidemiological-scale EIV sequences display A at site 384.

### Distinct consensus sequences circulated during the outbreak

We examined the consensus sequence for each horse to determine whether particular mutations had been fixed during the course of the outbreak. Within Newmarket, we observed only two different consensus sequences in the 19 yards sampled (excluding horse B03 with only six sequences, Table 1). A third consensus sequence was observed outside of Newmarket in the only horse sampled in yard T. Notably, variants carrying mutation G230A (Arg62Lys of the mature HA) were found in 14 horses that were stabled in 6 different yards, including all sampled horses from yard L. While 38 horses exhibited G230 at the consensus level, twelve horses exhibited A230. One horse (L27) that was sampled twice displayed G230 in the consensus on the first sampling date (April 10^th^) and A230 subsequently (April 14^th^). Despite detecting four different EIV consensus sequences, only a single mutation (A230) was fixed during the short time frame of the outbreak in Newmarket.

**Table 1:** Sequence diversity statistics for each sample.

Horse	Swab Date mm-dd	No. of seq.	Total no. of mutations	No. of stop codons	Mean pairwise distance ±SE	Mean d_N_/d_S_	Consensus
A01	03-13	67	21	2	0.0007±2.1E-05	0.62	G230
B02	03-25	32	8	0	0.0006±4.2E-05	1.28	G230
B03	03-25	6	6	0	0.0018±2.4E-04	1.27	A21
C04	03-26	63	30	0	0.0011±3.5E-05	0.92	G230
D05	03-27	82	8	0	0.0002±9.0E-06	0.57	G230
D06	03-27	40	3	0	0.0002±1.5E-05	1.39	G230
E07	03-27	68	6	1	0.0002±1.1E-05	0.43	G230
E09	03-28	50	22	0	0.0010±3.2E-05	1.45	G230
E10	03-28	80	40	0	0.0011±1.9E-05	0.98	G230
G08	03-28	39	15	1	0.0009±4.0E-05	0.41	G230
F11	03-28	11	14	0	0.0006±9.0E-05	0.14	A230
E13	03-31	45	18	0	0.0009±3.0E-05	0.81	G230
E14	03-31	9	4	0	0.0010±2.4E-04	0.14	G230
E15	03-31	14	4	0	0.0006±9.3E-05	0.43	G230
D12	03-31	39	5	1	0.0003±1.9E-05	10.79	G230
E18	04-01	74	91	0	0.0005±1.5E-05	0.49	A230
E19	04-01	74	7	0	0.0002±1.0E-05	0.57	G230
H16	04-01	27	17	0	0.0014±7.8E-05	1.21	G230
I17	04-01	47	16	1	0.0008±3.0E-05	1.92	G230
K22	04-02	65	21	0	0.0007±1.7E-05	0.82	G230
D20	04-02	46	9	0	0.0004±1.9E-05	1.26	G230
J21	04-02	10	4	0	0.0009±1.5E-04	19.89	G230
H23	04-04	46	15	0	0.0007±2.7E-05	0.51	G230
H24	04-04	67	21	0	0.0007±1.7E-05	1.04	G230
L25	04-10	64	98	2	0.0012±2.9E-05	1.14	A230
L25	04-08	44	57	0	0.0007±2.8E-05	2.07	A230
L26	04-09	1	1	0	NA	NA	G230
L27	04-10	81	9	1	0.0002±8.6E-06	15.06	G230
L27	04-14	16	18	0	0.0003±4.6E-05	19.12	A230
N28	04-09	50	21	0	0.0009±3.9E-05	0.68	G230
L30	NA	9	11	0	0.0005±1.0E-04	0.43	A230
M29	04-08	47	15	1	0.0007±2.8E-05	1.44	G230
M31	04-09	14	7	0	0.0011±7.9E-05	1.06	G230
M32	04-10	61	32	3	0.0012±2.7E-05	1.03	G230
O33	04-11	26	23	0	0.0020±8.3E-05	1.11	G230
N34	03-14	39	19	0	0.0010±3.7E-05	0.48	G230
N37	04-14	42	36	1	0.0019±5.8E-05	1.54	G230
R38	04-14	56	16	0	0.0006±1.9E-05	0.89	G230
Q36	04-14	62	88	0	0.0009±1.9E-05	1.21	A230
P35	04-14	20	6	1	0.0007±6.2E-05	0.65	G230
L39	04-15	45	53	0	0.0004±2.1E-05	2.52	A230
L40	04-17	73	145	0	0.0025±8.0E-05	0.33	A230
S41	04-17	77	100	0	0.0007±1.7E-05	0.31	A230
N45	04-22	35	12	0	0.0008±3.7E-05	1.91	G230
V46	05-08	72	26	1	0.0008±1.9E-05	1.22	G230
L42	04-19	65	83	0	0.0006±2.1E-05	2.01	A230
L42	04-21	86	118	2	0.0011±1.7E-05	0.63	A230
L43	04-19	21	29	0	0.0008±7.5E-05	0.29	A230
L44	04-19	3	4	0	0.0007±3.7E-04	17.71	A230
L47	04-21	15	19	0	0.0006±8.5E-05	1.27	A230
T48	04-22	31	85	0	0.0016±6.3E-05	0.38	T69T384
U49	04-28	23	7	0	0.0007±4.4E-05	0.56	G230
W50	05-08	47	12	0	0.0006±2.3E-05	0.79	G230

### Dynamics of EIV genetic diversity at the individual and population level

**Figure 2:**
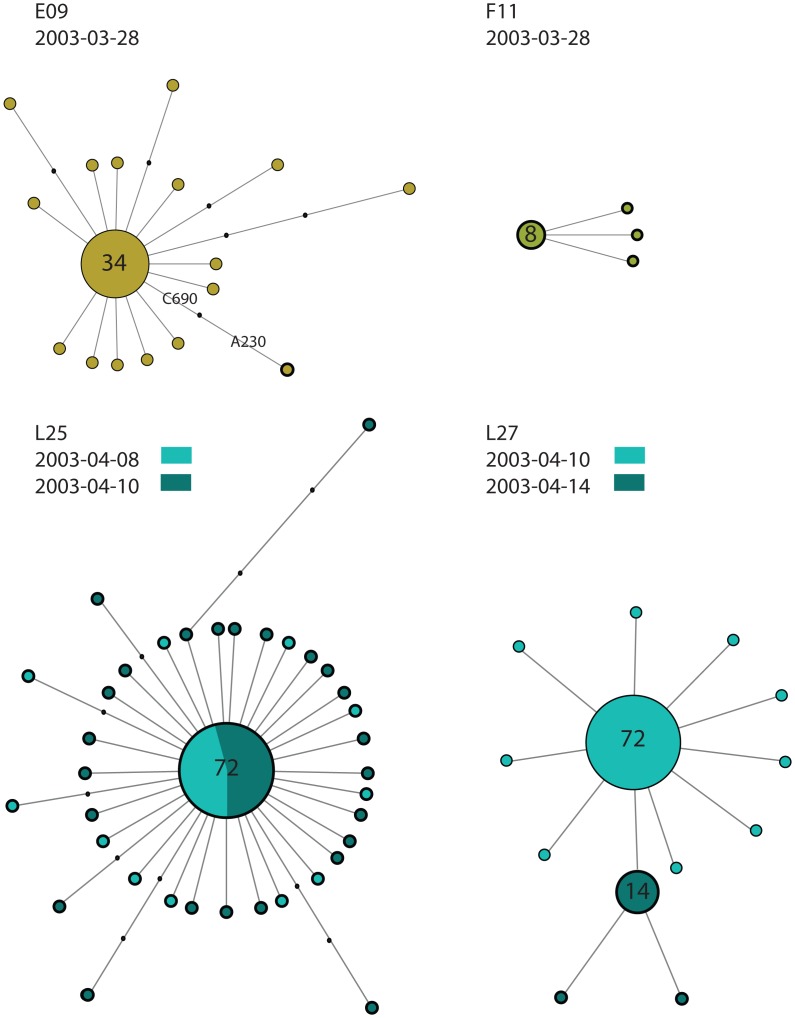
Median joining networks illustrating the intra-host viral diversity of four representative horses. The networks were generated from all the sequences from an individual horse and the size of the circle is relative to the sequence frequency. The color indicates the yard and day the sample was taken from. Sequences with A230 are circled with a thick line. Note that a single clone has A230 in horse E09. Black dots on the branch indicate the number of mutation differentiating two sequences.

At the individual level, all horses (except for L27 and L40 as discussed below), displayed viral populations characterized by the presence of a dominant variant and a number of minor variants. For example, Figure 2 illustrates the intra-host diversity among four horses with respect to sequences with G230 and A230: the first horse to have A230 as a dominant variant was F11 on March 28^th^, with all clones sequenced from this horse carrying the mutation. On the same day, horse E09 exhibited A230 linked to C690, suggesting that A230 could have been present in the viral population before March 28^th^. Of the three horses sampled twice during the course of the outbreak, horse L25 and L42 exhibited sequences with A230 as the dominant variant on both sampled days, whilst L27 initially displayed sequences with G230 and then A230 four days later (Figure 2) illustrating the individual heterogeneity in viral dynamics. This change in the dominant variant could be due to the latter variant superseding sequences with G230 or as a result of a mixed infection.

At the population level, we observed the asymptotic appearance of new mutations (Figure S2A) and an increase in mean pairwise distance (Figure S2D) mirroring the increase in the number of infected individuals (Figure S2B), suggesting that the viral diversity sequenced is not limited by the number of horses sampled (Figure S2C). The frequency of sequences with G230 fluctuated over the course of the outbreak, with an increase in sequences with A230 between April 15^th^ and April 22^nd^ (Figure S2E) and no sequences with A230 after April 22^nd^ suggesting the extinction of the sequences with A230.

### Evolution of EIV across scales

To determine how the genetic diversity of EIV during the outbreak relates to that observed at the global scale, we aligned all the unique intra-host sequences together with 284 publicly available epidemiological sequences (Dataset S2). The phylogeny of the unique sequences from our data set noting the yards in which the sequence was found is shown in Figure S3. As expected, with the exception of nine sequences from a single horse (see below and [2]), all sequences clustered together, showing that despite the high levels of genetic diversity observed at the individual level, they represent a minor component of the global genetic diversity of EIV. More surprising was the lack of phylogenetic differentiation between yards. The most commonly found sequence was G230 with 1235 copies in 38 different horses from 19 yards (Figure S3). Strains related to G230 continue to circulate in Europe and worldwide today. The second most frequently found sequence was A230, found in 490 copies from 14 horses in six different yards. This complete sequence is not found at the global scale and is likely to be a variant unique to this outbreak. As noted previously [2], one horse (L40) contained nine copies of a sequence similar to a strain from Florida Clade 1 circulating in North America and South Africa in 2003 (Figure S3 inset), suggesting a mixed infection of the Florida Clades 1 and 2. However, as we found no other horses carrying Florida Clade 1 sequences, these nine sequences were excluded from subsequent analyses.

### Minority variants are transmitted between horses and yards

To determine whether EIV transmission bottlenecks are sufficiently loose to allow the passage of multiple variants between horses during the outbreak, we examined the number of shared mutations between horses. Of the 493 mutations detected, 117 non-synonymous (24%) and 55 synonymous (11%) mutations were shared among different horses (Table S1). Of 117 non-synonymous mutations, 19 were located at antigenic sites and 6 at glycosylation sites, indicating that variants with distinct antigenicity could arise within single horses and be transmitted. Interestingly, two mutations causing stop codons were shared between horses: G585A was shared between L25 and M32, and G711A was shared between A01 and M29. G585A, which was linked to A230 in horse L25 and G230 in horse M32, likely arose independently as both horses were sampled on the same day. It was not possible to determine whether G711A was generated *de novo* in A01 and M29 or whether it was transmitted. Horses A01 and M29 were stabled in different yards and the period of time between sampling was 26 days.

In most cases only two horses shared a particular mutation, but we observed up to eight mutations shared between two horses (e.g. between L25 and L42). To determine whether mutations shared between horses followed a non-random pattern consistent with transmission pathways, we compared the distribution of observed shared mutations between pairs of horses to the null (random) expectation. The latter distribution was obtained by randomly assigning the observed mutations to individual horses. Results show that the pattern of shared mutations between pairs of horses was indeed non-random (Chi-squared test, p<0.001), indicative of inter-horse viral transmission.

Only seven of the 172 shared mutations were unique to a single yard and there were no significant differences in the number of shared mutations within yards compared to between yards when horses housed only in Newmarket were considered (W = 5614, p-value = 0.79). However, there was a significant association between the number of shared mutations and the distance between yards (slope = −1.14, p<0.05) and the number of days separating the dates of infection in the yards (slope = −0.03, p<0.001). Overall, these results suggest that a large number of mutations were shared between yards and the distance between yards and the time period between infection of horses correlates with the number of shared mutations.

### Mixed infections are common at the individual and yard level

**Figure 3:**
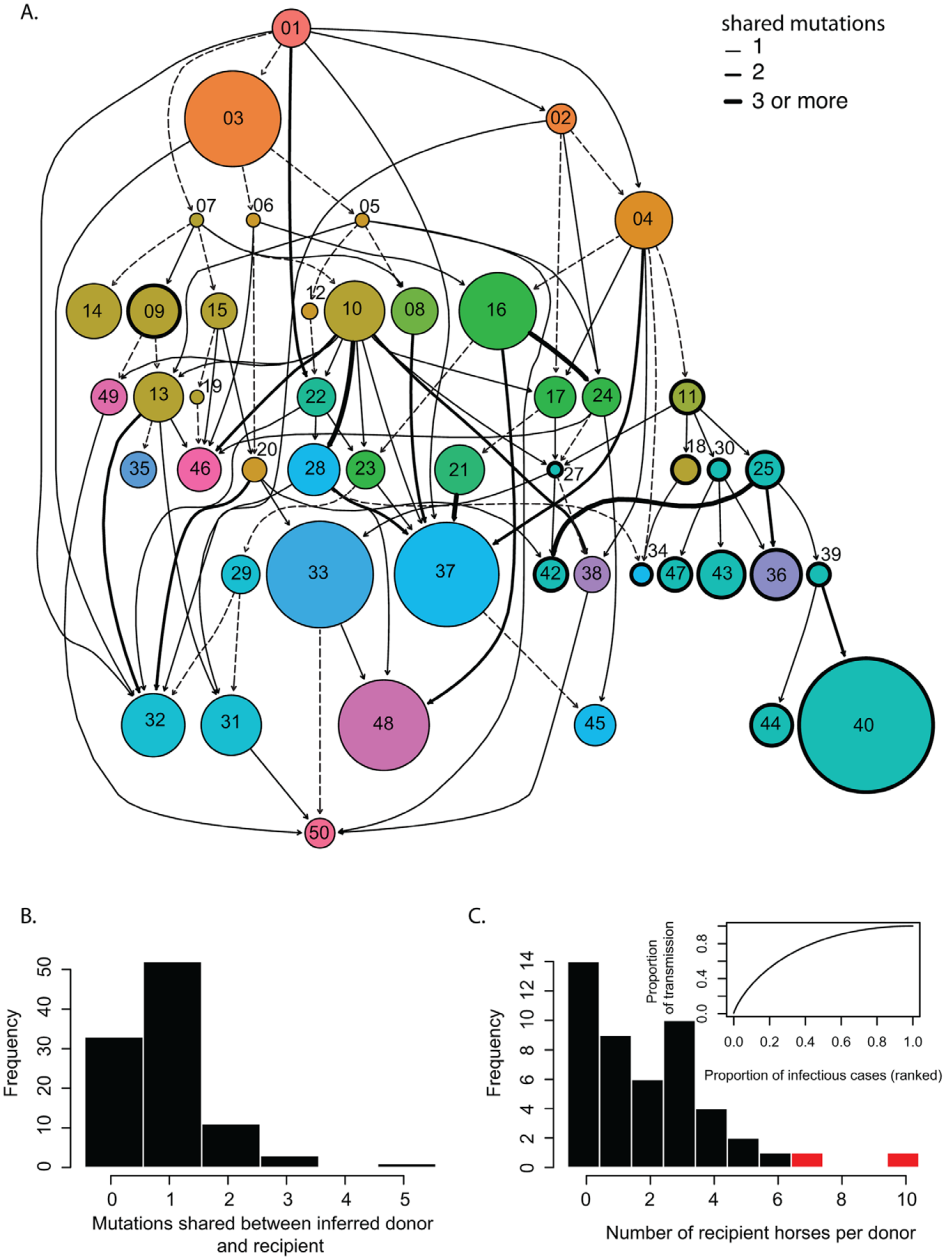
Reconstruction of EIV transmission pathways during the outbreak. (A) Transmission network inferred from the sequences, sampling date and locations for 48 horses. Each circle represents a horse colored according to training yard as in Figure 1. The size of the circle is proportional to the intra-host mean pairwise distance. Circles with thick black edges represent horses that have the A230 mutation. Arrows between circles represent inferred transmission events from the SeqTrack analysis. The corresponding number of mutations shared between any two horses is shown in the edge list in Table S2. Dashed arrows are for horses that only share the reference sequence. (B) Frequency distribution of the shared mutations between donor and recipient horses. (C) Distribution of the number of recipients per donor horse with the expected transmission caused by different percentage of cases (inset). The red bars represent the highly connected horses (E10 and the first sampled horse A01).

To test whether mixed infections are common in the field during an outbreak we used a graph based algorithm designed to reconstruct disease outbreaks using genetic data, sampling date and location [22] as phylogenetic approaches with few fixed mutations provide multifurcating trees with little resolution. Prior to this analysis, we removed 242 sequences that exhibited 81 mutations found linked to either A230 or G230, as such homoplasies are known to affect the parsimony-based inference of SeqTrack [22]. Hence our analysis was based on intra-host sequences derived from 48 horses from 19 yards for which both location and sampling dates were known (including two horses outside Newmarket). The inferred transmission events summarized per horse are shown in Figure 3A while the distribution of shared mutations between ancestor and descendant is shown in Figure 3B. The transmission network suggests that mixed infections are frequent, as 25 out of 48 horses exhibited common mutations with two or more other horses, including the two horses from outside of Newmarket (17 out of 48 if the reference sequence is excluded). In addition, we observed up to five shared mutations between horses, indicative of relatively wide transmission bottlenecks (Figure 3B and Table S2). As mutations introduced during the PCR amplification could bias these results, we repeated the analysis with sequences found at least twice in one host and which are highly unlikely to be the result of PCR errors. Although this conservative data set included only 40 mutations, it still suggested the presence of mixed infections (nine out of 37 horses) and loose bottlenecks, with up to five shared mutations (e.g., between L25 and L42 excluding three homoplasious mutations, not shown). We evaluated the compartmentalization between the yards by using the Slatkin and Maddison method [23], previously applied to the phylogeography of H5N1 IAVs [24]. The number of inter-yard transmissions (S = 442) based on the maximum likelihood phylogeny was lower than those expected from simulations (p<0.001) providing evidence of compartmentalization by yard. Additionally, transmissions from yard E to other yards occurred more frequently than expected by chance (Figure S4B). As expected based on sampling dates, transmissions from yards L to E were less frequent than expected despite both yards having the largest number of horses sampled (n = 10 and n = 8, respectively, Figure S4A). Hence, although there was an overall clustering by yard, there was also evidence of specific inter-yard mixed infections.

### Inferences from the transmission network

Additional analyses of the network revealed that horse E10 was most central (relative betweenness = 0.19, average = 0.045, SE = 0.008), requiring the fewest steps to access every other horse. As such, horse E10 is influential for the spread of the virus during the outbreak. Additionally, five other horses were critical for transmission to a subtree of the network (i.e., articulation points). For example, horse E07 was critical for transmission to E14, E09, E15 and E10 and horse L25 was critical for the transmission to L39, L44 and L40.

By fitting exponential random graph models to the directed network, we compared the graph to a random network as well as a network including the yard as an exogenous covariate. The latter model was a better fit to the data (AIC: 820.74, BIC: 826.46 versus AIC: 797.07, BIC: 808.52 with yard), suggesting that the yard a horse belonged to might have played a role in the transmission dynamics during the outbreak. Given the central role of some horses in the spread of EIV during the outbreak, we looked for evidence of superspreaders. Accordingly, the geometric distribution was the best fit to the data, very closely followed by the negative binomial; this provides limited evidence of potential superspreaders either as a result of the mode of transmission of EIV or the relatively small sample size of the study (Table 2 and Figure 3C) [25]. We also looked for individual factors associated with increased transmission. Accordingly, there was no significant relationship between the number of horses transmitted to and the age of the horse, time since last vaccination, the number of vaccine doses in the horses' lifetime and shedding load (n = 15 after removal of missing data).

**Table 2:** Model fitting to out-degree distribution according to [25].

Model	Parameters	CI (2.5%–97.5%)	AIC
Geometric	Mu = 2.08	1.70–2.51	188.50
Negative binomial	Mu = 2.08	1.53–2.83	189.09
	Size = 1.6	0.75–4.28	
Poisson	Mu = 0.32	0.25–0.40	204.35

### Epidemiological analyses

The effective reproductive number R_t_ is the number of secondary infections resulting from a single infectious individual at time *t* [26], and a crucial parameter in infectious disease epidemiology [26]. R_t_ can be estimated directly from the transmission network; that is, by determining who infected whom and thereby capturing individual variation in transmission. Although reconstructing transmission networks is practically difficult, we can use the pathways of transmission determined by sequence data.

**Figure 4:**
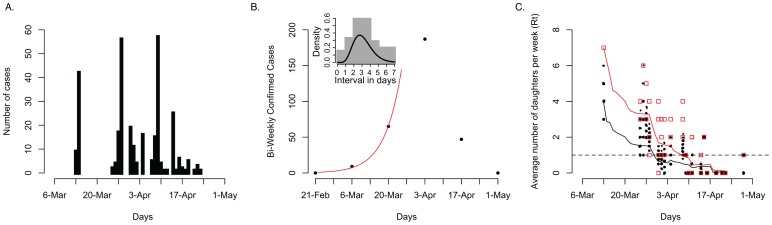
Transmission dynamics during the course of the outbreak. (A) Influenza cases in Newmarket between the 13^th^ of March 2003 to the 5^th^ of May 2003. (B) Exponential epidemic growth in Newmarket (R_t_ 1.8) with the inset showing the distribution of serial intervals from experimental data and a gamma distribution curve of shape 7.4 and scale 0.42 (Dataset S4). (C) The effective reproductive number, R_t_ measured from the epidemic trees generated from randomly pruning the transmission network. Dots indicate the number of secondary cases resulting from each primary case (random jitter was used to avoid superposition on the x and y axis). The black line represents a moving average with a window size of 14 days. The red squares represent the number of offspring per sampled horse according to the transmission network and taking into account mixed infections and the red line is a moving average of those numbers.

It is estimated that 1311 horses were kept in training in Newmarket at the time of the outbreak [14], of which 899 were tested and 306 were scored as infected during the outbreak (Figure 4A) with the highest number of infected horses (57 cases) detected on the 9^th^ of April. To calculate R_t_ at the start of the outbreak following [27], we fitted an exponential growth rate λ to the EI incidence using a generalized linear model with Poisson errors (see Methods). Converting λ using the probability distribution function of the serial interval provided an R_t_ estimate of 1.4–2.3 depending on the time-series and length used (weekly versus biweekly)(Figure 4B). Using the serial intervals derived from experimental data directly ([2] and Dataset S4), we obtained an R_t_ estimate of 1.8. Using the reconstructed transmission network, we estimated R_t_ over the course of the outbreak, which decreased from 3.9 to around 1.5 during the period of epidemic growth, before incidence declined and the epidemic went extinct (Figure 4C). Our estimate of R_t_ was slightly higher if mixed infections were taken into account but decreased faster (Figure 4C). While these estimates are broadly similar, the network approach may initially overestimate R_t_ because of under-sampling individuals early in the outbreak (Figure 4C), whereas the epidemic inference is relatively robust to sampling but it is subject to some uncertainty as the time-series is very short (Figure 4B). The network approach allows further dissection of transmission pathways including those that result in mixed infections (red and black lines in Figure 4C), which would not be apparent from incidence data alone.

## Discussion

### Comparison of within-host viral populations between experimentally and naturally infected horses

The genetic diversity of the HA1 gene described here resembles that described in previous intra-host studies of RNA viruses [2], [3], [28]. The sequences contained mutations at glycosylation or antigenic sites and mutations causing stop codons that were likely to be transmitted and these mutations could provide altered viral fitness and hence influence transmission dynamics [29]. The estimated mutation frequency was slightly higher than previous estimates [2], which may be attributed to differences in strains and horse immunity. Although erroneous inclusion of PCR and sequencing errors can be difficult to account for, by aligning sequences against a reference for nucleotide polymorphism detection and using a high Phred quality score for identification of these mutations, the number of false-positives is likely to be limited. Additionally, previous experiments quantifying mutations introduced by PCR errors suggest they only occur at a frequency of 1E^−6^/nt/cycle under our experimental conditions [2].

### The importance of sequencing beyond the consensus

The limited number of different consensus sequences obtained indicates that one sequence per horse would be insufficient to reconstruct the transmission dynamics during this outbreak. Although we have sequenced only HA1, it is likely that even a whole genome consensus sequence would still not be sufficient to reconstruct a transmission network, thereby highlighting the importance of intra-host sequencing data. This is in contrast to previous studies that successfully reconstructed the transmission dynamics of other RNA viruses, and notably Foot-and-Mouth-Disease virus, from consensus sequences per host or per farm [20], [30], [31]. The small number of consensus sequences is likely to be the result of the short time-scale of the outbreak and the short sequence analyzed. Most horses had only one dominant variant and a variable number of minor variants with one to nine mutations relative to the consensus sequence. However, for one horse (L27) sampled on multiple days, the dominant variant changed over the course of the infection (G230 followed by A230) either as a result of variant evolution or a mixed infection with a different variant. We have reported similar changes in within-host consensus sequences of swine influenza in pigs and canine influenza virus in dogs [3], [18].

The number of sequences and mutations shared between horses is indicative of relatively loose transmission bottlenecks in natural EIV infection, with up to three variants and eight mutations being shared between horses. However, it should be emphasized that inferred transmission events should be treated with caution given the limitations of the methodology used here (see Methods). In particular, although single mutations can be transmitted, it is extremely difficult in these cases to differentiate true transmission events from noise due to *de novo* mutations and/or laboratory artefacts. Clearly, transmission bottlenecks play a major role in shaping viral diversity, although these will vary in magnitude by virus and by mode of transmission. Indeed, Norovirus [32] and HIV [33]–[35] are characterized by strong genetic bottlenecks of transmission, with only a low proportion of variants being transmitted.

### Phylodynamics of EIV at the local scale

Taking Newmarket as a whole, the dominant viral population fluctuated over the course of the outbreak from G230 to A230 and back to G230. This change in the dominant lineage illustrates the speed with which lineages can originate, circulate and become extinct during outbreaks. Such genetic turnover may be due to changes in environmental conditions (such as temperature and relative humidity), varying densities of uninfected hosts and also changes in the immune status of the population during the outbreak, each of which will impose a distinct selection pressure on the virus. Alternatively, it could simply reflect the inherently stochastic nature of viral transmission.

The transmission network and the number of shared mutations between yards suggests that the spread of EIV in Newmarket was highly diffusive and that the training yard only played a minor role in structuring the viral population. Additionally, our data shows that EIV does not necessarily spread between horses sharing the same yard. Indeed, EIV was likely transmitted between horses that were kept in yards that were 2.7 km apart (yard E to L). Social networks may better explain part of the transmission dynamics especially as horses from different yards establish direct contact during their daily routines, and which may also explain the fluid dynamics seen in the transmission of human influenza virus [36], [37]. However, we have limited information on the contact network between horses and yards, which constrains further integrated social network analyses.

A small number of individuals are often disproportionately well-connected [38], with potentially important implications for virus transmission and control [25]. Such individual variation can both affect disease extinction as well as the frequency of outbreaks, often causing more explosive epidemics [25]. EIV exhibited limited heterogeneity in transmission efficiency with certain individuals appearing to be responsible for a large proportion of transmission events. For example horse E10 was likely to have been highly influential in the spread of EIV with ties to 10 other horses and was probably the main reason for the large number of transmissions from yard E. Other horses were critical for the transmission to subtrees of the network. Isolating such horses might have limited the spread of EIV during the outbreak. However, we found no factors that were significantly associated with individual transmission efficiency, although this may be due to the small sample size. Clearly, future research on understanding the factors (behavioural, genetic and/or immune history) that determine the variation in transmission between individuals would be useful in designing better control strategies. Also, greater sampling density would be necessary to conclude anything more firmly about the role of individuals in overall transmission.

Our estimates of R_t_ from sampled epidemic trees derived from the network showed time-dependent variation, rapidly decreasing during the period of epidemic growth. Given non-random under-reporting and under-diagnosis in both time and space during the outbreak, the reconstructed network is only partly known, which may influence the estimation of R_t_. On the other hand, R_t_ shows the transmission potential of mixed infections more fully. The incidence time-series was also relatively short, limiting the resolution of initial R_t_ estimates (∼1.8 but ranging from 1.4 to 2.3). While the direct estimate was relatively consistent with the network approach, it was lower than those from a recent outbreak in 2007 in Australia (2.04 in peri-urban and 1.99 in rural areas) [39] and an outbreak in racecourses across Japan in 1971 (2.08–5.02) [40]. These differences may lie in the immune status of the populations. The Australian outbreak affected naïve horses (Australia being an EI free country does not routinely vaccinate) and the Japanese outbreak took place before widespread vaccination was common practice, while vaccination coverage in the Newmarket horse population was universal as a result of compulsory racehorse vaccination. No official movement restrictions were instigated during the outbreak but vaccination was carried out soon after initial diagnosis [16]. Thus, the rapid decline in R_t_ may have been due to the depletion of susceptible horses either as a result of exposure or vaccination.

### Transmission dynamics at the yard level

Although a larger sample size will doubtless reveal more evidence on intra-yard transmission, its relatively low frequency in the current data suggests that the virus may be more routinely transmitted in other locations than the training yard and via other mechanisms than close contact. Despite the fact that horses spend a considerable amount of time in the yard, they can mix with horses from other yards during training, and the intensity of training conditions in racehorses might facilitate transmission. Moreover, intermediate hosts and fomites are likely to play a role in influenza inter-yard transmission. There is mounting evidence on EIV transmission over long distances, including wind-borne aerosol spread over 1–2 km in a recent outbreak in Australia [41]; and, the possibility of mechanical transmission by flies [42], [43]. Our results also suggest that implementing control measures within training yards would be of limited value considering the level of inter-yard transmission and the need to maintain training regimes for thoroughbreds. Control strategies such as movement restrictions to prevent further dispersal of horses incubating infection outside Newmarket might be more feasible at a regional scale.

### Mixed infections and their impact on transmission dynamics

One of our most striking observations is the frequency and range of mixed infections. The mixed infection of viruses from different clades, as in L40 [2], was probably a consequence of the long distance transportation of horses following the global racing circuit. In 2003 alone, L40 competed in races in the UK, France and Germany, which might explain how the mixed infection of two divergent EIV lineages could have occurred. Interestingly, L40 tested positive for EIV by ELISA or qPCR eight out of ten times between April 15^th^ and May 8^th^, suggesting that it could have been infected continuously for a 23-day period, possibly as a consequence of a mixed infection. Apart from this case, the intra-host genetic diversity and the network reconstruction suggest that approximately 52% of the sampled horses had mixed infections of closely related variants (the exact number of transmitted variants cannot be estimated, particularly for sequences that differ on one nucleotide where *de novo* mutations and PCR artefacts cannot be ruled out). Even when we used a “conservative” data set (i.e. only sequences that had mutations in multiple clones in a horse), we detected evidence of mixed infections (24%), suggesting that this observation is unlikely to be the result of PCR or sequencing artefacts.

Mixed infections could be the result of co-infections (i.e., a heterogeneous viral population resulting from a single transmission event) or super-infections (i.e., a mixed infection resulting from multiple independent transmission events). In most cases, it is not possible to differentiate between these mechanisms, although for a co-infection to be feasible a super-infection must have occurred in an individual earlier in the transmission chain. Mixed infections are clearly the main pre-requisite for intrasubtypic reassortment, shaping viral diversity during the course of an outbreak and impacting on the global evolution of EIV [12]. Indeed, reassortment may be occurring frequently among lineages that are circulating within outbreaks, although our focus on HA1 meant that we could not detect this process.

**Figure 5:**
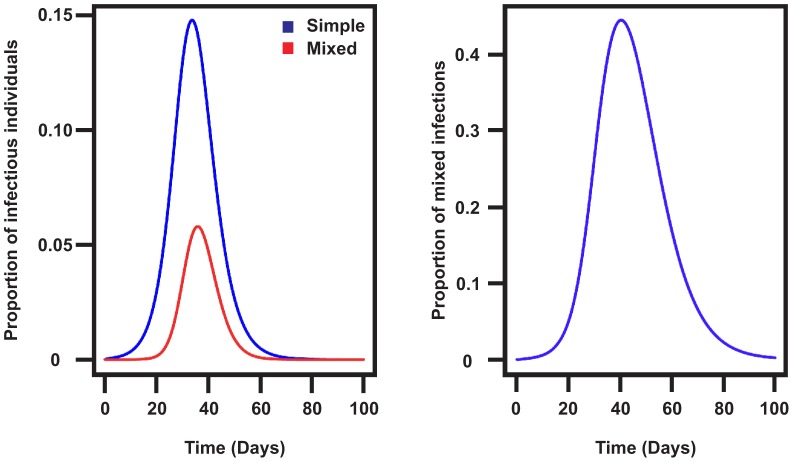
The impact of mixed infections in the SEIR model. (A) Proportion of infectious individuals in the basic SEIR model and the modified model. (B) Proportion of reinfections in the Newmarket vaccinated population using data from experiments with heterologous vaccination for the latent and infectious periods [15].

Mixed infections can have important implications for epidemiological models (Text S1). Modifying a simple SEIR model suggested that the level of reinfection is highly dependent on the latent and infectious periods. If the latent and infectious periods are long, like in a naïve population [44], very rapidly almost all infections will involve mixed infections. If vaccination results in shorter latent and infectious periods [15], then fewer infections will be mixed (40% in the parameterization in Figure 5), consistent with our finding of 52% of horses with mixed infections. Epidemic size also depends on whether infectious periods for mixed infections are longer or shorter (i.e. longer infectious periods would result in larger epidemics). Interestingly, the two horses with mixed infections for which we have repeated nasal swabs tested positive for EIV for seven (L27) and 23 days (L40), much longer than the average three days observed in *in vivo* studies. Despite the fact that these observations are consistent with our hypothesis, a bigger sample size is necessary to support it and repeated sampling of affected individuals during an outbreak will be required to clarify this issue. Overall, our findings suggest that the prevalence of mixed infections detected using genetic data should be considered when investigating IAV dynamics as it could affect the epidemic size.

## Materials and Methods

### Generation of viral sequence data

Nasal swabs were collected during the outbreak between March and May 2003 [14]. We analyzed a total of 199 nasal swabs from individual horses during the outbreak. Details of shedding and date of sample collection are listed in Dataset S5.

RNA extraction, viral quantification by real-time PCR and PCR analysis were performed as described in [2]. Briefly, we used the QIAmp viral RNA mini kit following the manufacturer's instructions to extract viral RNA from nasal swabs (280 µl initial volume). We used Superscript III (Invitrogen) and primers Bm-M-1 and Bm-HA1 [45] to generate cDNA of the M and HA genes, respectively. We performed qPCR using the QuantiTect Probe PCR kit (Qiagen) with fluorogenic hydrolysis type probes according to the manufacturer's instructions and using the same primers and probe as in [2]. All samples, no template controls, positive and negative controls and plasmid standards were run in triplicate for each run. We used Platinum Pfx (Invitrogen) and primers Bm-HA1 and EHA1007rw to amplify HA1 as described in [2]. We used the QIAquick Gel Extraction kit to gel purify PCR products, which in turn were cloned using the Zero-Blunt TOPO PCR Cloning kit for sequencing (Invitrogen) following the manufacturer's instructions. Clones were sequenced using fluorescent sequencing chemistry and ABI 3730×l capillary sequencers at the Wellcome Trust Sanger Institute.

The sequences from each clone were then trimmed and assessed for quality prior to assembly. Only contigs >903 nt were used for subsequent analyses. Contigs were aligned against a consensus HA1 sequence of A/equine/Newmarket/5/2003 using a bioinformatics tool for mutation detection from viral sequences (Ramirez-Gonzalez, Hughes, and Caccamo, in preparation). This provides basic statistics relating to the number of stop codons, number of synonymous and non-synonymous mutations, and location of mutations (antigenic site, receptor binding site) for each sequence. Nucleotide variants were considered real if their Phred score was greater than 25. Nucleotides with a Phred score below that value were considered identical to the consensus nucleotide. Sequences containing high-quality insertions or deletions that altered the reading frame were counted.

### Sequence analysis

A total of 2361 sequences were generated, of which 2222 were from horses in Newmarket. Yards were denoted A to W (Figure S1). Individual samples were identified based on the yard ID and a horse number (i.e. sample A01 was derived from horse 1 in yard A). Mutations were annotated according to mutation type (synonymous, non-synonymous), whether they were in glycosylation sites, antigenic sites or receptor binding sites. The sequence diversity within each host was measured as the pairwise uncorrected distance (p-distance) and the number of synonymous (d_S_) and non-synonymous (d_N_) nucleotide substitutions per site (ratio d_N_/d_S_), which was also estimated for the data set as a whole. d_N_/d_S_ was estimated using the Single Likelihood Ancestor Counting (SLAC) algorithm available in the HyPhy software package [46] with sequences with stop codons excluded. To determine whether the number of shared mutations between pairs of horses was non-random, mutations were randomly assigned to the same number of horses the mutation was found in and the assignment was repeated 100 times. Subsequently, the number of pairs of horses sharing randomly assigned mutations was compared to the observed data using a Chi-squared test.

To analyse sequences at the epidemiological scale, 283 sequences were collated from GenBank (Dataset S2). Sequences from the outbreak were aligned with the publically available data using MAFFT [47]. The phylogeny was reconstructed using the maximum likelihood (ML) method available in RAxML [48], employing the GTRGAMMA model with 500 bootstrap replicates. Further, the yard for each sequence in the ML phylogeny was assigned to the tips as a single character and we used the Slatkin-Maddison test [23] to determine the extent of gene flow between the viral populations in each yard. This test was implemented in PAUP* 4.0 [49] to determine the number of transmission events between yards using the DELTRAN parsimony optimization algorithm. To assess the uncertainty of our reconstruction of the minimum number of inter-yard transmission events, we randomized the yards on the tree tips 1000 times and repeated the parsimony optimization. The results were summarized to determine whether transmission events between yards inferred from the maximum likelihood tree were greater than the frequency of the same events if the localities were randomly distributed.

### Network analysis

SeqTrack was used to trace the spatiotemporal dynamics of EIV across Newmarket [22], [50]. SeqTrack is a graph based algorithm the reconstructs the most parsimonious genealogy from genetic data. It is particularly suitable to infer transmission pathways during disease outbreaks, where samples typically display low levels of genetic diversity. As the algorithm does not take into account multiple sequences from the same host, we reconstructed the transmission network from the SeqTrack adjacency matrix for each horse and subsequently for each yard. The reconstructed network was compared to a random network with equal nodes and edges as well as a network including the training yard as a covariate using exponential random graph models [51], [52]. We used the approach of [25] to fit offspring distributions (poisson, geometric and negative binomial) to the inferred transmission network using maximum likelihood. A generalized linear model with Poisson errors was used to determine whether the number of out-degrees could be explained by the age of the horse, the time since last vaccination, the number of vaccine doses or the shedding load of the horse. The centrality of each horse was determined using relative betweenness centrality [53], [54]. This is a measure of the influence the horse has in the transmission network as it counts the number of steps that are required for it to infect every other horse in the network. Articulation points were identified within the network [55]. All analyses were performed using the R software environment [56] and the network was drawn using the graph [57] and Rgraphviz packages [58], [59].

### Epidemiological analyses

The outbreak investigation, sampling protocols and the method used for establishing a positive diagnosis of equine influenza virus were previously published [14]. R_0_ was estimated directly from the infectious histories of 899 horses tested over the course of the outbreak. The date of infection was determined based on the first positive ELISA or the date when the viral copy numbers was above 150 copies per microliter (due to the limits of false positive detection in qPCR), whichever occurred first. The intervals between infections were determined based on the time period between positive nasal swabs determined by qPCR from different experiments including [2]. These experiments were based on natural transmission of H3N8 in naïve and vaccinated horses (heterologous or homologous vaccination) (Dataset S4). We combined the data from these studies as the intervals between infections did not vary significantly between studies (F_3,19_ = 3.27, P = 0.8). We fitted Poisson, geometric and gamma distributions to these intervals using maximum likelihood. A gamma distribution (mean = 3.3 days, variance = 1.3) provided the best fit and had the lowest AIC, thus was used for further calculations.

We estimated the initial growth rate of the epidemic (λ) by fitting an exponential curve to incidence data using a generalized linear model with Poisson errors. We explored a range of intervals for fitting, up to and including peak incidence; the short time-series limited the fitting procedures for the shorter intervals but by peak incidence, the rate of epidemic growth had potentially been curtailed. We converted the estimated growth rate to measure R_0_ (initial R_t_) using the serial interval from the transmission experiments.

To calculate R_t_ over the course of the epidemic as in [60], we used a resampling approach from the network to select a single donor from possible lists of candidates with equal probability to generate 100 epidemic trees. The number of secondary cases per infected horse inferred from each tree was calculated and averaged across the possible epidemic trees to provide a time varying estimate of R_t_ and compared to the estimate from the network (i.e., taking into account mixed infections).

The implications of the mixed infections were addressed by modifying the classical SEIR framework to estimate the frequency of potential mixed infections and the impact of the epidemic (Text S1).

## Supporting Information

Dataset S1 List of accession numbers from this study. The horse identifier and geo-location is provided as latitude and longitude in decimal degrees.
(TXT)

Dataset S2 Alignment of 284 publically available HA1 sequences from equine H3 viruses. The sequences were aligned using MAFFT and the sequences were trimmed and gaps were removed to agree with the sequences in this study.
(FASTA)

Dataset S3 Spatio-temporal spread of EIV in Newmarket. KML file viewable in GoogleEarth to visualize the spatio-temporal spread of EIV in Newmarket.
(KML)

Dataset S4 Intervals in days between significant shedding between donor and recipient horses. This data was generated from three experimental datasets of naturally transmitting horses. The horses were either vaccinated with a homologous or heterologous vaccine and challenged with A/Newmarket/5/2003 or A/Newmarket/1/1993.
(TXT)

Dataset S5 Viral shedding values per yard.
(CSV)

Figure S1 Map of the yard locations in Newmarket. Yards are represented by squares and sampled yards are in red with capital letters (A to W). The number of horses in training within the yard is shown in brackets. Yard S, O, T and W are not shown because the location of yards S is unknown and yards O, T and W are outside of Newmarket. The date of first infection of each yard is shown on a graphical timeline with each unit representing a day. Major roads in Newmarket are shown in orange.
(PDF)

Figure S2 Dynamics of viral diversity during the course of the outbreak. (A) Cumulative increase in observed new mutations, (B) cumulative number of cases in Newmarket, (C) number of horses sampled for each time point, (D) cumulative mean pairwise distance over the course of the outbreak, (E) number of sequences with G230 and A230 mutations during the course of the outbreak.
(PDF)

Figure S3 Maximum likelihood phylogenetic tree for HA1 segment clones from samples obtained from infected horses during the outbreak. The tree is rooted on A/equine/Kentucky/5/02. Branch lengths are drawn to scale. The circles represent different yards from which the clones were obtained and are colored according to Figure 1. The number of sequences with G230 and A230 are represented by pie charts for each horse and colored according to the training yard. The inset phylogeny shows the outbreak sequence data (boxed) within the context of the global phylogeny. Light blue represents the pre-divergence lineage, blue the Eurasian lineage, purple the American lineage, red the sublineage Florida Clade 1, and green represents sequences from the Florida clade 2 sublineage. The arrow indicates the position of the nine sequences from L40 as reported in [2].
(PDF)

Figure S4 Transmission dynamics at the yard level. (A) Transmission network summarized according to yard. The circles represent training yards and the size is relative to the number of horses sampled in each yard. Dashed arrows are for yards that only share the reference sequence. For all other arrows, the number of shared mutations is shown within black boxes on the arrow. Transmission events within a yard are shown with a curved arrow. Yards that have the A230 mutation are shown with thicker edges. (B) Frequency of character changes from one yard to another determined by the mapping of yards as a character onto the phylogeny from RAxML. The red points represent the observed frequency of unambiguous character change. The boxplot represents the summary from 1000 permutations (dark horizontal segment shows the median, the box surrounds the first and third quartiles, whiskers represent the 95% bounds and black points mark outliers). The observed character changes (red points) outside of the 95% bounds of the simulations (whiskers) represent significant transmission pathways.
(PDF)

Figure S5 Compartmental SEIR model allowing for mixed infections.
(PNG)

Figure S6 Estimation of the proportion of reinfection for two scenarios; using parameters from Glass et al. (unvaccinated population based on the 1963 emergence of the H3N8 sub-type) and Baguelin et al. (from the 2003 outbreak in Newmarket using data from experiments with heterologous vaccination for the latent and infectious periods).
(PNG)

Figure S7 Impact of a different length of infectious period for individuals with mixed infections. In red is the infectious profile without reinfections, the black curve is the total number of infectious individuals in the mixed infection model and the blue curve is the number of individuals with mixed infections.
(PNG)

Table S1 List of all shared mutations. The table provides information on the mutation type (synonymous, non-synonymous, in a glycosylation site, in an antigenic site, present at the epidemiological scale, homoplastic).
(TXT)

Table S2 Edgelist showing the donor and recipient horses and the shared mutations.
(TXT)

Text S1 A theoretical study of the disease dynamics with mixed infections.
(PDF)
