# Supplementary figures and images for: Transmission of Equine Influenza Virus during an Outbreak Is Characterized by Frequent Mixed Infections and Loose Transmission Bottlenecks
Source: PLoS Pathog. 2012 Dec 20;8(12):e1003081. doi: 10.1371/journal.ppat.1003081 (PMC3534375; doi:10.1371/journal.ppat.1003081)

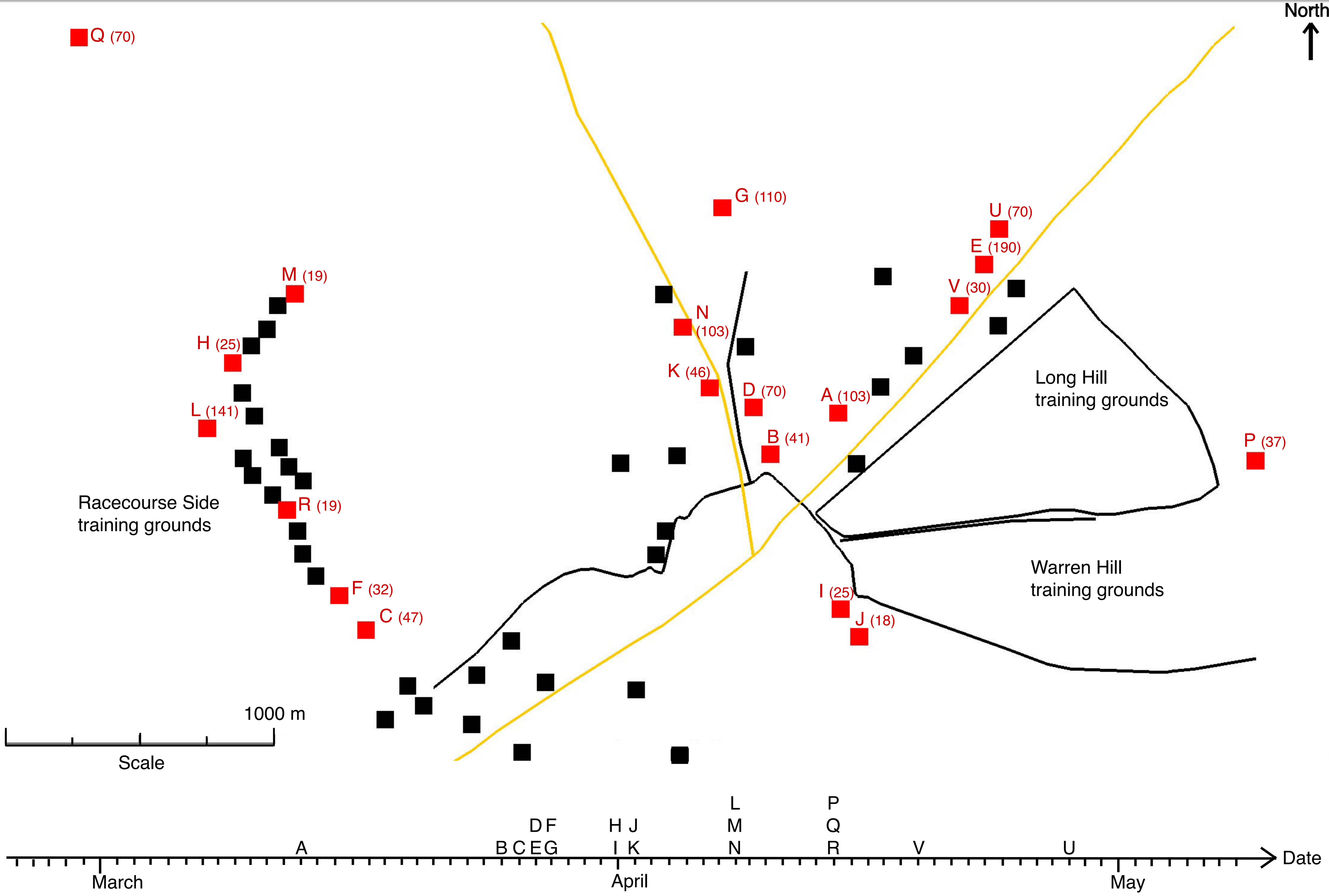

Supplement: Figure S1 — Map of the yard locations in Newmarket. Yards are represented by squares and sampled yards are in red with capital letters (A to W). The number of horses in training within the yard is shown in brackets. Yard S, O, T and W are not shown because the location of yards S is unknown and yards O, T and W are outside of Newmarket. The date of first infection of each yard is shown on a graphical timeline with each unit representing a day. Major roads in Newmarket are shown in orange. (PDF) [file ppat.1003081.s006.pdf]

Figure S2.

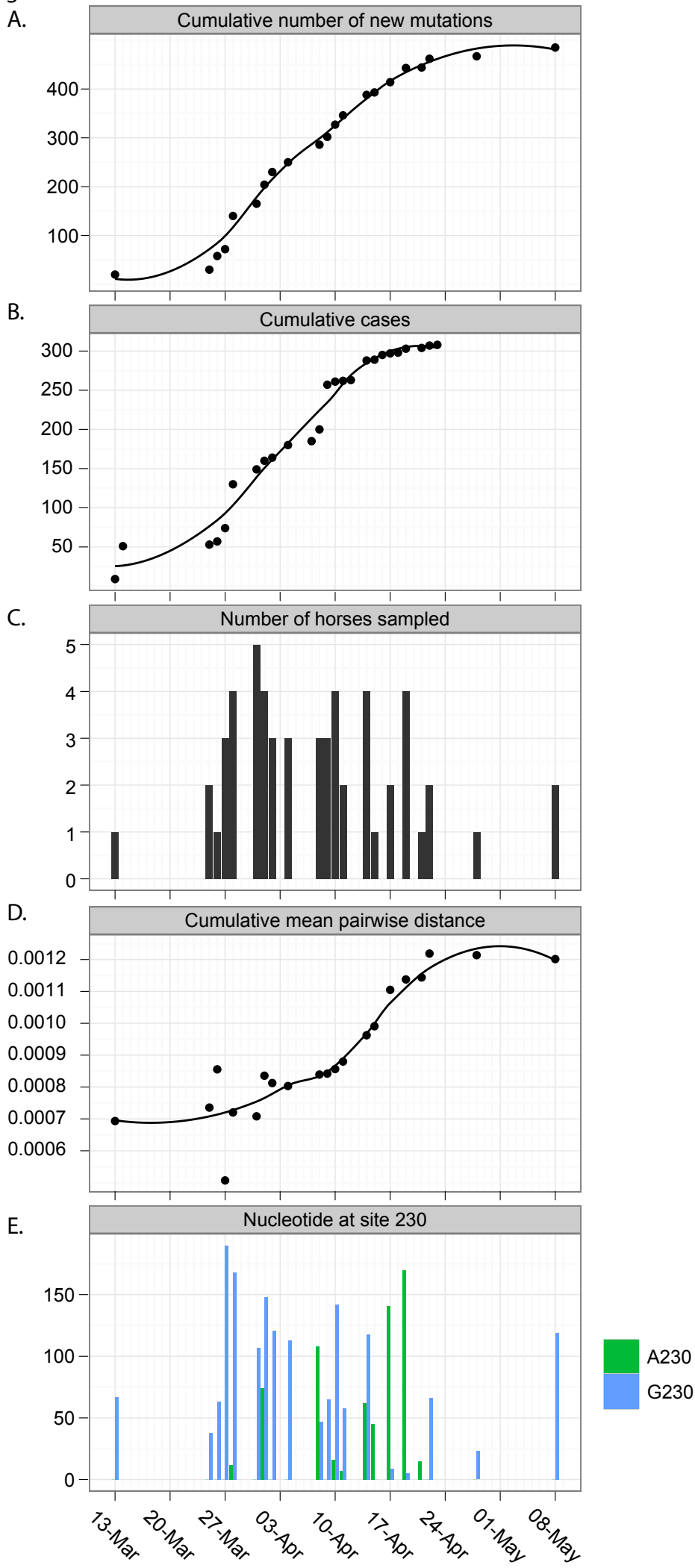

Supplement: Figure S2 — Dynamics of viral diversity during the course of the outbreak. (A) Cumulative increase in observed new mutations, (B) cumulative number of cases in Newmarket, (C) number of horses sampled for each time point, (D) cumulative mean pairwise distance over the course of the outbreak, (E) number of sequences with G230 and A230 mutations during the course of the outbreak. (PDF) [file ppat.1003081.s007.pdf]

Figure S3.

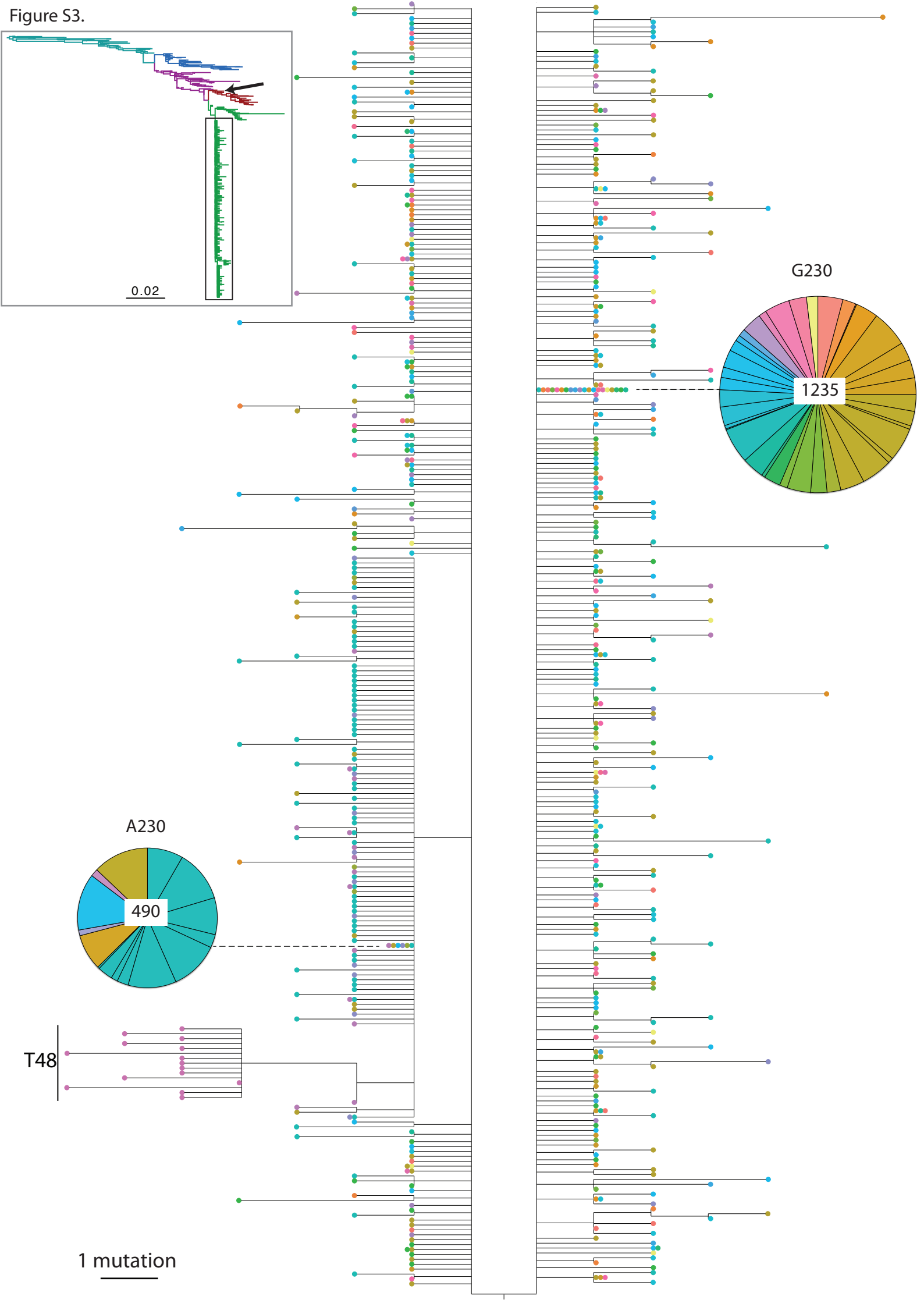

Supplement: Figure S3 — Maximum likelihood phylogenetic tree for HA1 segment clones from samples obtained from infected horses during the outbreak. The tree is rooted on A/equine/Kentucky/5/02. Branch lengths are drawn to scale. The circles represent different yards from which the clones were obtained and are colored according to Figure 1. The number of sequences with G230 and A230 are represented by pie charts for each horse and colored according to the training yard. The inset phylogeny shows the outbreak sequence data (boxed) within the context of the global phylogeny. Light blue represents the pre-divergence lineage, blue the Eurasian lineage, purple the American lineage, red the sublineage Florida Clade 1, and green represents sequences from the Florida clade 2 sublineage. The arrow indicates the position of the nine sequences from L40 as reported in [2]. (PDF) [file ppat.1003081.s008.pdf]

Figure S4.

A.

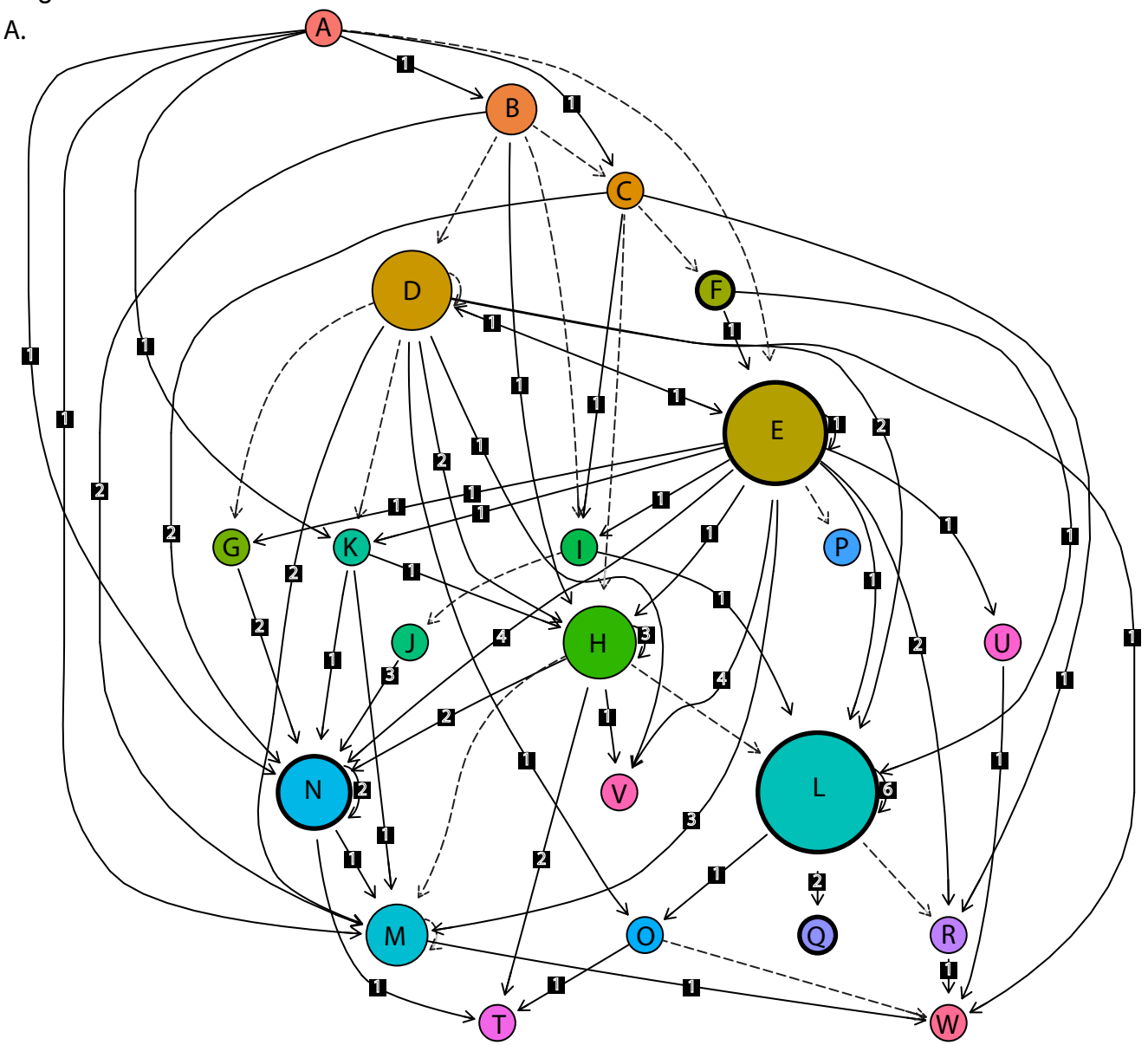

B.

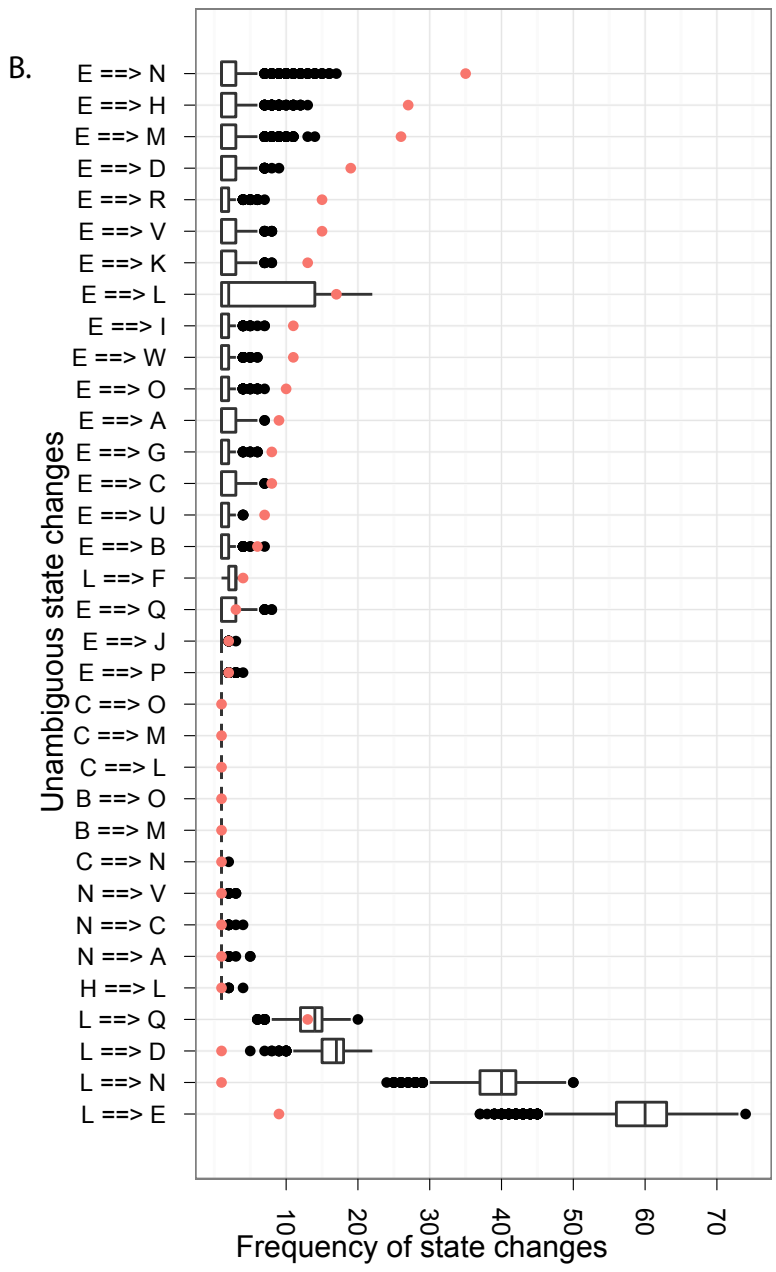

Supplement: Figure S4 — Transmission dynamics at the yard level. (A) Transmission network summarized according to yard. The circles represent training yards and the size is relative to the number of horses sampled in each yard. Dashed arrows are for yards that only share the reference sequence. For all other arrows, the number of shared mutations is shown within black boxes on the arrow. Transmission events within a yard are shown with a curved arrow. Yards that have the A230 mutation are shown with thicker edges. (B) Frequency of character changes from one yard to another determined by the mapping of yards as a character onto the phylogeny from RAxML. The red points represent the observed frequency of unambiguous character change. The boxplot represents the summary from 1000 permutations (dark horizontal segment shows the median, the box surrounds the first and third quartiles, whiskers represent the 95% bounds and black points mark outliers). The observed character changes (red points) outside of the 95% bounds of the simulations (whiskers) represent significant transmission pathways. (PDF) [file ppat.1003081.s009.pdf]

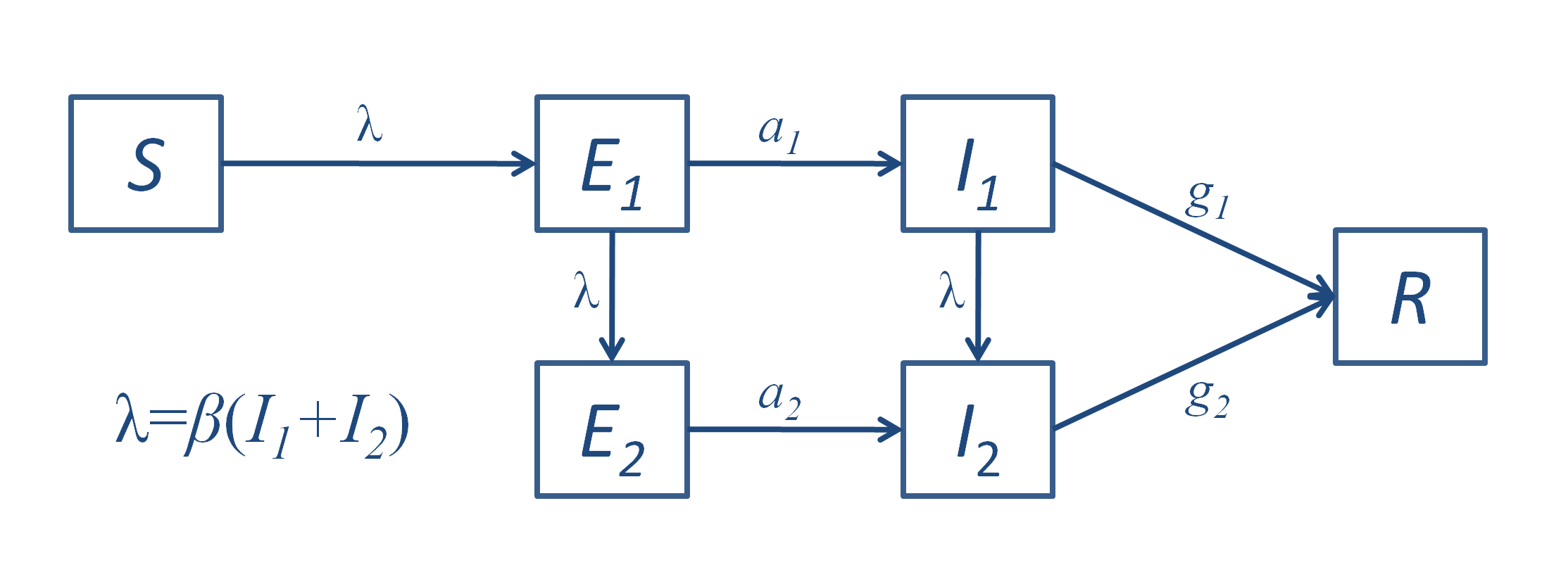

Supplement: Figure S5 — Compartmental SEIR model allowing for mixed infections. (PNG) [file ppat.1003081.s010.png]

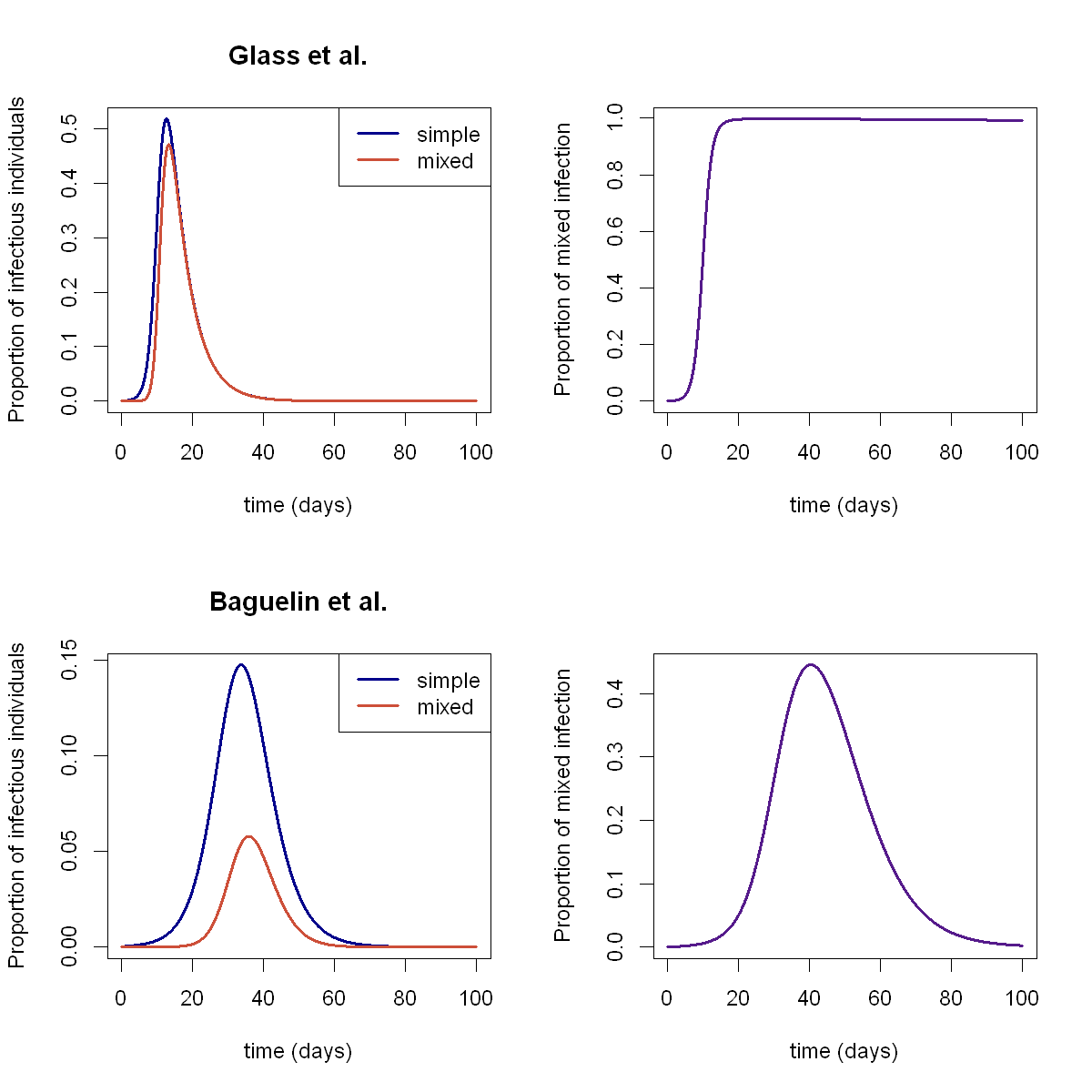

Supplement: Figure S6 — Estimation of the proportion of reinfection for two scenarios; using parameters from Glass et al. (unvaccinated population based on the 1963 emergence of the H3N8 sub-type) and Baguelin et al. (from the 2003 outbreak in Newmarket using data from experiments with heterologous vaccination for the latent and infectious periods). (PNG) [file ppat.1003081.s011.png]

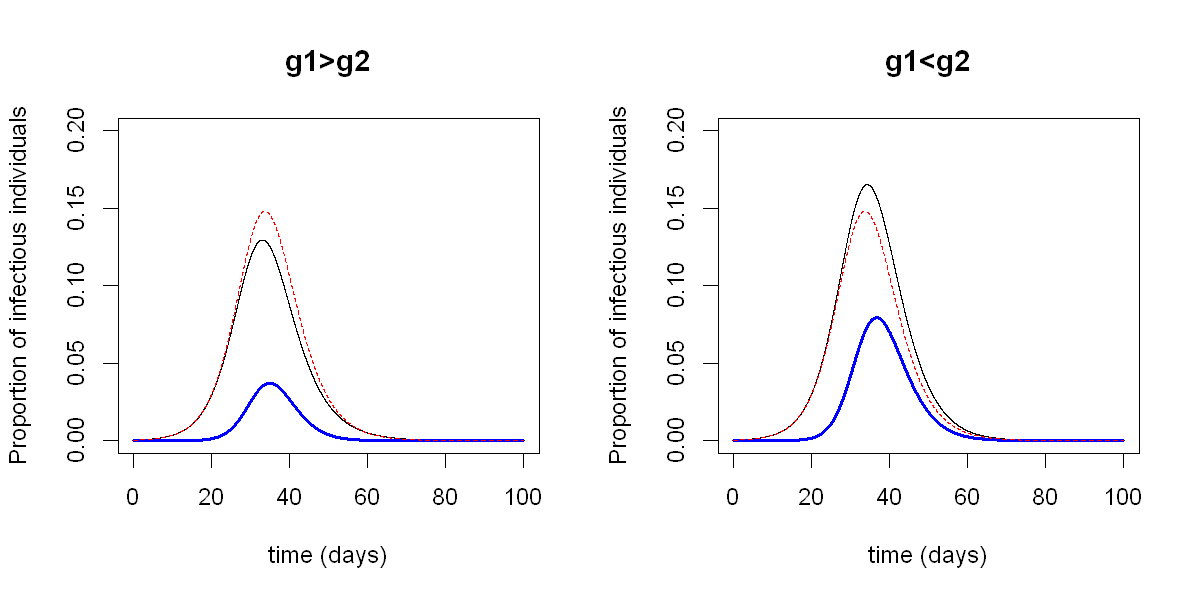

Supplement: Figure S7 — Impact of a different length of infectious period for individuals with mixed infections. In red is the infectious profile without reinfections, the black curve is the total number of infectious individuals in the mixed infection model and the blue curve is the number of individuals with mixed infections. (PNG) [file ppat.1003081.s012.png]
