# Supplementary material for: Transmission of Equine Influenza Virus during an Outbreak Is Characterized by Frequent Mixed Infections and Loose Transmission Bottlenecks
Source: PLoS Pathog. 2012 Dec 20;8(12):e1003081. doi: 10.1371/journal.ppat.1003081 (PMC3534375; doi:10.1371/journal.ppat.1003081)
Supplement: Text S1 — A theoretical study of the disease dynamics with mixed infections. (PDF) [file ppat.1003081.s015.pdf]

Disease dynamics with mixed infections: a theoretical study for Spatio-temporal analysis of intrahost genetic diversity of equine influenza virus H3N8 during an outbreak reveals frequent mixed infections and loose bottlenecks

1 Background and methods

We modify the classical SEIR framework in order to model mixed infections during influenza transmission. Our aim is to estimate using relatively simple assumptions and from a purely dynamical point of view the frequency of potential mixed infections and the impact on their dynamics of disease.

1.1 The simple SEIR model

Most models of influenza transmission at a population level are based on compartmental models derived from the SEIR model. The framework is based on the following assumptions:

- the probability that a susceptible individual becomes infectious is proportional to the number or proportion of infectious individuals in contact with it;
- once an individual is infected, it cannot be re-infected.

Mathematical formalisms can be used to translate this process in order to derive quantitative results. Using ordinary differential equations (ODEs), i.e. a deterministic, compartmental and time-continuous model, the classic SEIR model can then be written as:

$$\begin{cases} \dot{S} = -\lambda S \\ \dot{E} = \lambda S - aE \\ \dot{I} = aE - gI \\ \dot{R} = gI \end{cases} \quad (1)$$

with $\dot{X} = \frac{dX}{dt}$, $\lambda = \beta I$ the force of infection, $1/a$ the latent period and $1/g$ the infection period.

1.2 The SEIR model allowing for mixed infections

We now assume that exposed (E or I) individuals can be reinfected by circulating viruses, allowing for two additional compartments, by splitting the E and I compartments respectively into E_1/E_2 and I_1/I_2, with the index 1 and 2 noting repectively the individuals with simple and mixed infections.

A schematic view of the new model is given in Figure S5 and the system can be described by the following system of ODEs:

$$\begin{cases} \dot{S} = -\lambda S \\ \dot{E} = \lambda S - (a_1+\lambda)E_1 \\ \dot{E} = \lambda E_1 - a_2 E_2 \\ \dot{I} = a_1 E_1 - (g_1+\lambda)I_1 \\ \dot{I} = a_2 E_2 + \lambda I_1 - g_2 I_2 \\ \dot{R} = g_1 I_1 + g_2 I_2 \end{cases} \quad (2)$$

with $\lambda = \beta(I_1+I_2)$, the force of infection, $1/a_1$ and $1/a_2$ the latent period for the simple and mixed infections, and $1/g_1$ and $1/g_2$ the infectious period for the simple and mixed infections.

1.3 Choice of parameters

We based our choice of parameters on two studies [1, 2] which calculated explicitely influenza transmission by fitting transmission models to two outbreaks of equine influenza in New York State (1963) and Newmarket (2003). Of the two, one is set in a totally naive population [2] and the other in a partially immunised population [1]. In the two studies, the latent and infectious period were estimated from other data sources.

It is unclear how mixed infections will modify the latent and infectious periods. We first kept $a_1=a_2$ and $g_1=g_2$ to measure the extent of mixed infections during an epidemic. We subsequently allow the infectious period to vary to study the impact of a change of infectious period on the overall dynamics.

Paper	β	$1/a$ (days)	$1/b$ (days)	R_0
Glass et al.	1.85	1.25	5.5	10.85
Baguelin et al.	0.78	2.17	3.3	2.6

Table A: Parameters used for the model of mixed infections

2 Results

2.1 Probability of escaping multiple infection

Assuming a constant force of infection over a short interval of time, it is possible to estimate the probability that an infected individual escaped multiple infections:

$$P(avoid\ multiple\ infection) \approx \frac{a_1}{\lambda+a_1}\frac{g_1}{\lambda+g_1}$$

this means that the probability of a multiple infection depends only on the force of infection and the balance between the latency and recovery rates. Multiple infections should be more common near the peak of infection when λ is maximized.

Using the two sets of parameters from Table A, we observe a high level of reinfection. In the naive population, given a high rate of transmission and long latent and infectious periods (short latency and rapid recovery rates), very rapidly nearly all infections involve mixed infections. In the vaccinated population, a large proportion of infected individuals experience mixed infections, culminating around the peak of the epidemic, where more than 40% of the infectious cases are carrying mixed infections (Figure S6).

2.2 Impact on the dynamics of transmission

If $a_1=a_2$ and $g_1=g_2$ then (2) is equivalent to (1), as it can be simplified using $E=E_1+E_2$ and $I=I_1+I_2$. When $g_1 \neq g_2$, the initial phase of the epidemics remains the same as in the case without re-infections, but then when a population with mixed infections arises the overall dynamics changes. If $g_1>g_2$ (infectious periods are shorter for mixed infections) then the size of the epidemics decreases, giving final size values that would be observed with a smaller R_0 value. On the contrary, if $g_1<g_2$ (longer infectious periods for mixed infections) then the size of the epidemics increases, giving final sizes values that would be observed for a higher value of R_0 (see Figure S7).

3 Discussion

- These results support the observation of mixed infections being common during equine influenza outbreaks.
- This model can be adapted to study more detailed level of infections.
- We use a time-continuous and deterministic model for simplicity. For small populations though, at a yard level, stochasticity is expected to play an important role.
- The values of the latent and infection periods were based on values observed experimentally. These values might not translate directly to values under natural transmission. If infection follows an experimental inoculation, the time of infection will be known, but the quantity of virions used for the inoculation might affect the transmission dynamics (e.g. inducing a shorter latent period). On the other hand, an observation derived from transmission experiments does not allow precise assessment of when transmission actually occurs. Additionally, the sensitivity of the test used to assess infectivity in experiments can affect the reliability of these measurements.
- The transmission parameter λ is directly related to R_0 the number of secondary cases arising from the introduction of a single infectious individual in a fully susceptible population. Measurement of R_0 is difficult, although it is linked with the initial growth rate (assuming a fully susceptible population). But the structure of the population implies that the values of R_0 at the yard level will be different from the one at the population level (see [1).
- If infectious periods for mixed infections seems to have a big impact on the number of re-infections and the overall epidemic size (Figure S7). In fact, altered infectious periods do not so much change the overall number of infections than the presence of individuals with mixed infections at a given time. More individuals with mixed infections are likely to be sampled if mixed infections involve a longer period of shedding.

. References

. [1] Baguelin, M, Newton, J. R, Demiris, N, Daly, J, Mumford, J. a, & Wood, J. L. N. (2010) Journal of the Royal Society, Interface / the Royal Society 7, 67–79.

. [2] Glass, K, Wood, J. L. N, Mumford, J. A, Jesset, D, & Grenfell, B. T. (2002) Epidemiology and infection 128, 491–502.
